# Microalgal Synthetic Biotechnology: From Genetic Models to Deployable Photosynthetic Chassis

**DOI:** 10.34133/research.1449

**Published:** 2026-10-19

**Authors:** Jin Liu

**Affiliations:** School of Resources and Environment, Center for Algae Innovation & Engineering Research, Engineering Research Center of Watershed Carbon Neutrality of Ministry of Education, State Key Laboratory of Food Science and Resources, Nanchang University, Nanchang 330031, China.

## Abstract

Microalgae have long been considered promising platforms for producing biochemicals and supporting environmental applications. Realizing this potential will require turning microalgal species into predictable and reusable engineering platforms. However, it remains challenging to engineer such evolutionarily diverse, compartmentalized photosynthetic eukaryotes in a predictable way, because their behavior depends strongly on organelle organization, gene-expression context, and cultivation conditions. Here, we focus on 3 well-developed but functionally distinct chassis. *Chlamydomonas reinhardtii* provides the strongest genetic and cell-biological foundation among green microalgae; *Phaeodactylum tricornutum* serves as the main diatom system for genome engineering and systems-level pathway analysis; and *Nannochloropsis* spp., especially *N. oceanica* and *N. gaditana*, are more production-oriented and are particularly important for lipid engineering. We assess these chassis from the perspective of practical engineerability, including transformation and genome access, expression design, precise editing and regulatory control, and iterative design–build–test–learn workflows. We then discuss 4 representative trait areas: photosynthetic productivity and stress tolerance, storage lipids and omega-3 fatty acids, terpenoids and carotenoids, and pollutant removal. Despite substantial progress, the field is still limited by unstable transgene expression, weak portability of genetic parts, incomplete understanding of intracellular pathway organization, and poor agreement between laboratory phenotypes and process performance. Progress will require genetic design to be evaluated together with chassis physiology, cultivation performance, product recovery, and deployment-specific biosafety.

## Introduction

Microalgae are sources of proteins, lipids, pigments, and specialty chemicals, and possess potential for environmental applications [1]. Their capacity to convert light and CO_2_ into biomass and metabolites also makes them candidates for photosynthetic biomanufacturing [1,2]. In synthetic biotechnology, however, a chassis is useful only when engineered phenotypes can be interpreted, reproduced, and improved across design cycles [3]. Microalgae make this requirement difficult because they are diverse, compartmentalized photosynthetic eukaryotes rather than simplified bacterial or yeast hosts [2,4]. Their metabolic pathways span several organelles, so pathway output can change with protein targeting, metabolite transport, light regime, nutrient status, and growth phase [3,5]. Design rules developed in one lineage therefore often fail in another.

Recent work has expanded the microalgal engineering toolkit. Several species now support regulatory design, multigene expression, targeted genome editing, organelle engineering, and omics-guided pathway analysis [2,3,5,6]. These tools have broadened the agenda from photosynthetic carbon fixation to specialized metabolites and pollutant transformation. However, progress is uneven across microalgal lineages. The green microalga *Chlamydomonas reinhardtii* has the deepest genetic and cell-biological foundation, but it is not generally a production host [7]. Several industrially promising microalgae, on the other hand, remain difficult to engineer routinely because they lack reliable genetic tools [8–11]. An engineering strategy should therefore be evaluated in the chassis where it is used, not only by the trait or tool it targets.

Previous reviews have covered microalgal cell factories [2,3,5], engineering technologies [6,12–14], and major product classes [15–19]. A product- or tool-centered structure can hide a recurring problem: The same design can behave differently in different microalgae. This review therefore uses 3 reference chassis rather than products alone. *C. reinhardtii* remains the most genetically tractable green algal model, with strong depth in photosynthesis, cell biology, and molecular genetics [7]. *Phaeodactylum tricornutum* is the leading diatom system for combining genetic engineering with omics-guided and systems-level analysis [14,16]. *Nannochloropsis* spp., particularly *N. oceanica* and *N. gaditana*, are more closely linked to lipid- and eicosapentaenoic acid (EPA)-oriented production, while recent pigment-engineering studies extend its chassis value beyond native oleaginous traits [20,21]. These microalgae provide useful reference systems for comparison, rather than interchangeable examples.

Other microalgae also have substantial biotechnological value. For example, *Haematococcus pluvialis* and *Dunaliella salina* are well-established commercial sources of astaxanthin and β-carotene, respectively [22,23], while *Chlorella*, *Chromochloris*, and *Euglena* species are being developed for producing protein, pigments, and carbohydrates [8,24,25]. Their exclusion from the anchor chassis does not imply their limited importance. Instead, it reflects the focus of this review on species that combine well-developed genetic toolkits, increasing mechanistic knowledge, and feasible iterative engineering. The docosahexaenoic acid-producing stramenopiles such as *Schizochytrium*-like organisms perform no photosynthesis and thus are not considered here.

This review is organized around a simple premise: A microalga becomes a useful chassis only when genetic manipulation can be connected to reproducible, interpretable, and eventually deployable phenotypes. We therefore ask which microalgae are only genetically tractable, which design rules can be shared across algal lineages, and which limits remain tied to cell architecture or physiology. We also consider what evidence is needed before a promising laboratory phenotype can be regarded as production or application relevant. With these questions in mind, we first compare the developmental trajectories and current capabilities of *C. reinhardtii*, *P. tricornutum*, and *Nannochloropsis* spp. We then discuss the main layers of practical engineerability, including cell and genome access, expression architecture, precise editing and regulatory control, and iterative design–build–test–learn (DBTL) workflows. Finally, 4 representative trait areas, namely, photosynthetic productivity and stress tolerance, storage lipids and omega-3 fatty acids, terpenoids and carotenoids, and pollutant removal, are used to examine how chassis properties shape engineering outcomes.

## Three Reference Chassis for Microalgal Synthetic Biotechnology

Microalgal engineering does not yet have a single host that plays the role of *Escherichia coli*, *Saccharomyces cerevisiae*, or mature mammalian cell platforms. Methods for genetic manipulation, physiological interpretation, and cultivation practices still vary by species. Work has therefore clustered around species that can be manipulated with enough reliability to support repeated experiments. Among them, *C. reinhardtii*, *P. tricornutum*, and *Nannochloropsis* spp. give 3 reference points for this comparison [7,16,20]. Figure 1 places these systems along a trajectory from model establishment and genome resource development to tool expansion and trait-oriented engineering.

**Fig. 1.**
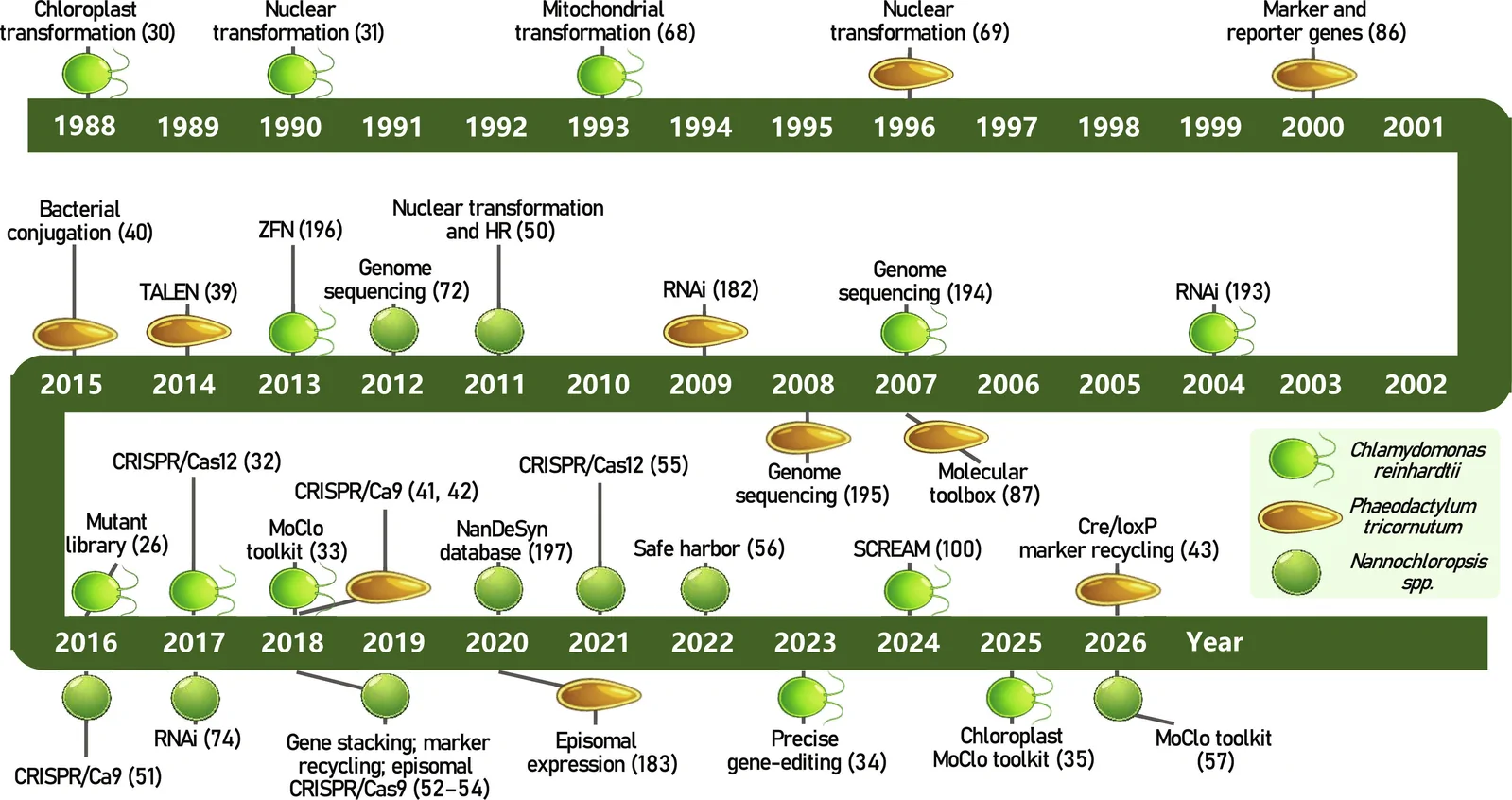
Developmental timeline of 3 reference microalgal chassis. The timeline highlights major milestones in genome-resource establishment, genetic-tool development, and trait engineering for *Chlamydomonas reinhardtii*, *Phaeodactylum tricornutum*, and *Nannochloropsis* spp. Relevant references are indicated in parentheses in the figure [26,30–35,39–43,50–57,68,69,72,74,86,87,100,182,183,193–197].

### *C. reinhardtii*: A genetically interpretable reference chassis

*C. reinhardtii* has the most extensive foundation in microalgal genetics and cell biology. Decades of research have characterized vegetative growth, inducible sexual cycle, photosynthesis, chloroplast function, flagellar biology, nutrient signaling, nuclear gene expression, and organelle communication [26–29]. In this context, engineered phenotypes in *C. reinhardtii* can often be linked to known cellular processes rather than described only in terms of changes in growth, fluorescence, or metabolite levels.

Also, *C. reinhardtii* has been well developed with nuclear and chloroplast transformation, promoter and intron design, mutant libraries, CRISPR/Cas-based editing, and increasingly precise gene-replacement strategies [26,28–36]. Therefore, it is widely used to investigate and engineer traits associated with photosynthesis, carbon acquisition, and light-responsive metabolism. Nevertheless, silencing remains a limitation in *C. reinhardtii*, and nuclear transgene expression often exhibits substantial clone-to-clone variation [37]. Its physiology and carbon-allocation patterns also differ greatly from those of oleaginous microalgae and diatoms. Therefore, a construct that performs well in *C. reinhardtii* may not work elsewhere. The species is best regarded as the field’s most developed system for mechanism-oriented engineering, rather than as a chassis for applications.

### *P. tricornutum*: Engineering within a diatom cellular context

The diatom *P. tricornutum* is characterized by a diploid genome, a complex plastid evolutionary history, distinctive membrane organization, and metabolic connections that differ markedly from those of green algae [38]. These features influence protein targeting, metabolite exchange, lipid assembly, pigment biosynthesis, and the cellular consequences of pathway engineering. The genetic toolkit for *P. tricornutum* now includes stable nuclear transformation, episomal expression, targeted mutagenesis, multiplex editing, and marker recycling [39–43]. In parallel, transcriptomic, proteomic, metabolic, and genome-scale studies have made the species a central system for linking diatom physiology with engineering design [14,16,41,44–46].

It is worth noting that regulatory elements, targeting peptides, and pathway locations that work in a green microalga cannot be assumed to behave similarly in a diatom. Therefore, engineering in *P. tricornutum* requires explicit attention to lineage-specific regulation, trafficking, and compartmentation [14,46,47]. This makes the species valuable for systems-guided pathway design, fucoxanthin-related biology, lipid remodeling, and studies of organelle interactions [14]. Its genetic parts and engineering methods, however, remain less standardized than those in *C. reinhardtii*, and performance can vary with strain and experimental context.

### *Nannochloropsis* spp.: A production-oriented oleaginous host

Species of *Nannochloropsis*, characterized by a compact haploid genome, are well known for their capacity to accumulate lipids and produce the long-chain polyunsaturated fatty acid (PUFA) EPA [48,49]. Among *Nannochloropsis spp*., *N. oceanica* and *N. gaditana* represent the most engineered species and have been developed with genetic techniques such as homologous recombination, CRISPR/Cas-based editing, marker recycling, multigene expression, and modular DNA assembly [50–57]. These features make *Nannochloropsis* spp. important hosts for studying carbon partitioning, lipid metabolism, EPA biosynthesis, and pigment–lipid co-production [21,48,49,58].

In *Nannochloropsis* spp., lipid engineering is now judged by productivity and product quality as well as lipid content [21,48]. Therefore, high lipid accumulation needs to be assessed together with growth. For EPA engineering, total EPA is only one measure. Its distribution among lipid classes and its stability during cultivation and downstream processing also determine product value [59]. The same principle applies to multigene engineering, because a construct has practical value only if expression persists during extended culture [52,60]. As a result, pathway engineering in *Nannochloropsis* spp. is being linked to lipid partitioning, cultivation performance, and product specifications [21].

This application-facing role distinguishes *Nannochloropsis* spp. from chassis developed mainly for mechanistic studies. Recent work has shifted from total lipid content toward pathway bottlenecks, subcellular desaturation and elongation, EPA partitioning, and combined production of fatty acids and carotenoids [52,57,59–62]. Predictability remains limited by species- and strain-dependent editing efficiency, a still-limited set of validated regulatory parts, and limited long-term or scale-relevant testing of engineered traits [55,57]. *Nannochloropsis* spp. is therefore best viewed as an advanced production-driven chassis with a growing toolkit, but its platform behavior still requires stricter benchmarking.

### Chassis choice as a determinant of engineering outcome

The comparison of these 3 chassis makes the host dependence of microalgal engineering explicit. *C. reinhardtii* is the most genetically interpretable model of green microalgae. *P. tricornutum* represents a well-established model diatom, in which the engineering design should confront lineage-specific organelle architecture and protein targeting. *Nannochloropsis* spp., on the other hand, stands out for its oleaginous and production-facing features and has been developed with versatile genetic tools. These are not interchangeable hosts but distinct engineering contexts (Table 1).

**Table 1.** Comparative positioning of the 3 reference microalgal chassis used in synthetic biotechnology

Chassis	Phylogenetic context	Engineering strengths	Principal limitations	Representative enabling advances	Best-fit applications
*Chlamydomonas reinhardtii*	Green alga; the deepest nuclear and chloroplast genetic model	Extensive genetic tools and genome resources; organelle transformation; strongest mechanistic basis for photosynthesis, CCMs, and cell biology	Nuclear transgene silencing and position effects; strong clone-to-clone variation; imperfect translation from model studies to production	MoClo assembly; mapped mutant libraries; DNA-free and precise CRISPR editing; high-throughput chloroplast engineering	Mechanism-first engineering; photosynthesis and CCM research; synthetic-circuit testing; terpenoid and carotenoid prototyping
*Phaeodactylum tricornutum*	Diatom; diploid nucleus; red-lineage complex plastid surrounded by 4 membranes	Leading diatom systems-biology framework; episomal expression and conjugative delivery; multiplex and DNA-free editing	Lineage-specific regulatory parts; relatively few broadly benchmarked parts; biallelic editing with careful validation	Stable nuclear and episomal expression; DNA-free multiplex editing; marker recycling; omics-guided and genome-scale pathway analysis	Systems-guided pathway design; lipid remodeling; fucoxanthin and EPA production; comparative studies of trafficking and compartmentation
*Nannochloropsis* spp.	Eustigmatophyte; compact haploid genome; native oleaginous and EPA-producing platform	High lipid and EPA productivity; feasible HR; multigene stacking; expanding CRISPR toolkit; strong production relevance	Locus- and strain-dependent editing efficiency; few validated safe harbors and parts; limited long-term and process-scale testing	HR and marker recycling; episomal CRISPR; Cas12a multiplexing, CRISPRi, and CRISPRa; modular part toolkit and safe-harbor expression	Storage lipids; EPA and designer oils; carotenoid-lipid co-production; application-oriented strain engineering and integrated biorefinery

CCM, carbon-concentrating mechanism; EPA, eicosapentaenoic acid; HR, homologous recombination

Reviews organized by method clarify transformation systems, CRISPR tools, assembly methods, and engineering strategies [2,3,5,6,13], while reviews organized by product give detailed accounts of lipids, carotenoids, terpenoids, and other outputs [15,17,18,58,63,64]. The 3-chassis-centered view adopted here takes a different perspective and discusses how genetic accessibility, cellular organization, and application context shape the strengths and limitations of each chassis.

## Genetic Tools and Engineering Workflows in Microalgae

The feasibility of a microalgal chassis depends on a set of genetic capabilities that work reliably together. DNA delivery, expression control, genome editing, and phenotype assessment are often discussed separately, but in practice, they are closely connected. Therefore, the following sections consider these capabilities as components of a shared engineering workflow rather than as independent tools.

### Transformation and genome access

Transformation is the first practical barrier in microalgal engineering. Glass-bead agitation, electroporation, particle bombardment, *Agrobacterium*-mediated transfer, and bacterial conjugation represent established DNA-delivery routes for microalgae [6,28,47]. The appropriate method depends on the species, cell envelope, and organelle target.

Glass-bead agitation works well for the transformation of *C. reinhardtii* when the cell wall is enzymatically removed by autolysin or a cell-wall-deficient mutant strain is employed [31,65]. Electroporation allows much higher transformation efficiency and works for both cell-wall-deficient and cell-walled *C. reinhardtii* strains [66,67], which is particularly useful for high-throughput screening and generation of mutant libraries [26,29]. These 2 methods are routinely used for nuclear transformation of *C. reinhardtii*. For chloroplast or mitochondrion transformation, particle bombardment is used [30,68]. In *P. tricornutum*, particle bombardment and electroporation have been well developed to deliver DNA for efficient nuclear transformation [69–71]. Polyethylene glycol-mediated transformation has also been established for *P. tricornutum*, provided that the algal cells were enzymatically treated to remove the cell wall [71]. Besides, bacterial conjugation has been developed to enable episomal DNA delivery into the *P. tricornutum* nucleus where the episome is maintained and replicated independently [40]. Compared to *C. reinhardtii* and *P. tricornutum*, *Nannochloropsis* cells are smaller and possess a more rigid cell wall, thus requiring much higher voltage for nuclear transformation [50,72,73]. This electroporation-based method has been well deployed to deliver DNA into *Nannochloropsis* nucleus for random or targeted integration, or for independent replication as an episome, which allows efficient gene overexpression, knockout, knockdown, or CRISPR/Cas-based editing [50–53,55,57,74]. The transformation efficiency is cell-phase dependent and can be considerably improved by using cells synchronized in the G2/M phase [75]. Particle bombardment has been reported in *Nannochloropsis* spp., for example, *N. salina*, but it is much less commonly used than electroporation for routine nuclear transformation [76].

These differences determine which engineering workflows are feasible after DNA delivery. *C. reinhardtii* offers broad access to the nucleus and chloroplast for transformation, as well as large-scale genetic screening. In *P. tricornutum*, diatom-adapted bacterial conjugation enables episome-based pathway construction and genome editing. As for *Nannochloropsis* spp., electroporation has become the main method for routine nuclear engineering and is increasingly used for product-oriented strain development. The useful question is therefore not only how efficiently DNA enters the cell, but what strain-engineering workflow the method makes possible.

### Expression architecture and part performance

After DNA delivery, engineering success depends on expression architecture, not promoter strength alone. In microalgae, the same coding sequence can behave differently with changes in regulatory parts, codon usage, insertion site, chromatin environment, or subcellular targeting signals [2,37]. The model alga *C. reinhardtii* illustrates this point well. In this alga, the *HSP70A-RBCS2* chimeric promoter is widely used because the *HSP70A* promoter region improves expression relative to *RBCS2* alone [77]. *RBCS2* introns are also central to many expression cassettes: Early work showed that endogenous introns, particularly *RBCS2* introns, could enhance foreign gene expression, and later studies demonstrated that intron-containing synthetic genes or systematic intron insertion can improve nuclear expression [78–80]. GC content and codon usage also influence nuclear transgene expression in *C. reinhardtii* considerably [81], so heterologous genes also require codon optimization. Besides, terminator choice can impact transgene expression output when paired with different promoters [82]. These studies explain both the strength of the *C. reinhardtii* expression toolbox and its sensitivity to construct architecture.

Nuclear transformants of *C. reinhardtii* often vary substantially in expression level because transgenes may integrate at different genomic sites and are exposed to different chromatin environments. The studies of transgene silencing, epigenetic silencing pathways, and targeted integration into safe-harbor loci all suggest that high-level expression depends not only on cassette strength but also on the genomic context of insertion [37,83–85]. The identification of the light-harvesting complex II chlorophyll *a*/*b*-binding protein M1 gene (*LHCBM1*) locus as a safe harbor for high transgene expression is particularly important because it shifts the problem from random screening of high-expressing clones toward more predictable insertion-site design [83]. Thus, in *C. reinhardtii*, part performance depends on both regulatory design and the genomic context of integration.

Expression engineering in *P. tricornutum* has developed along a different path from that in green algal models. Early transformation studies mainly used fucoxanthin chlorophyll a/c-binding protein (FCP) promoters and terminators for selectable-marker and reporter expression [69,86,87]. Promoter options were later expanded to include regulatory sequences from constitutively expressed endogenous genes and diatom-infecting viruses [88,89]. As an alternative to nuclear integration, the episomal expression system was established, featuring a plasmid capable of self-replication in the *P. tricornutum* nucleus [40]. This system allows stable transgene expression and helps address the position effect associated with random genomic integration [42,90]. The diatom has a red-algal-derived complex plastid surrounded by 4 membranes; thus, the nucleus-encoded plastid proteins have to be directed by diatom-specific signal peptides [91].

In *Nannochloropsis* spp., early genetic tools included endogenous promoters, reporter tags, antibiotic resistance markers, and multigene vectors that were used to engineer the EPA biosynthesis pathway [52]. Later, gene-stacking tools were developed, allowing assembly of multiple genes in a single plasmid for pathway engineering [92]. These tools are important because many target traits in *Nannochloropsis* spp., such as EPA synthesis, triacylglycerol (TAG) assembly, and carotenoid production, require coordinated expression of multiple enzymes instead of single-gene overexpression [52,60]. More recently, a modular toolkit has been developed, which expands regulatory parts and genetic elements and establishes a standardized assembly syntax for multi-gene expression and pathway testing in *N. oceanica* [57]. Notably, promoter performance in this microalga can be strongly affected by the paired terminator [57].

Insertion site and subcellular targeting are becoming increasingly important in *Nannochloropsis* spp. In *N. oceanica*, targeting an expression cassette to the nucleolar rDNA region could provide a genomic safe harbor for strong and consistent transgene expression; this system combines RNA polymerase I-driven transcription with an internal ribosome entry site to support translation [56]. For localization, in vivo fluorescent protein-based studies in *N. oceanica* established targeting routes to several cellular compartments and provided a practical reference for testing putative signal peptides [57,93]. This information is especially relevant because many *Nannochloropsis* engineering targets are compartmentalized and require enzymes to be localized to the appropriate cellular compartment for accessing substrates and cofactors [60].

Collectively, the value of a microalgal part library lies not simply in the number of available promoters, terminators, tags, or other genetic elements, but in whether these parts give predictable expression when tested across designs, independent clones, and growth states. All 3 chassis now support useful expression engineering, but the rules that govern stable and properly localized expression remain largely chassis-specific.

### Toward precise and controllable genome editing

Pathway engineering requires editing workflows that can make defined changes, recover lines without unwanted genetic scars, and work at different genes or genomic sites [94,95]. By these criteria, the 3 reference chassis differ in editing maturity.

Genome editing in *C. reinhardtii* has progressed from gene disruption to more defined genetic changes. DNA-free Cas9 ribonucleoprotein delivery enabled disruption without exogenous DNA [96]. CRISPR/Cas12a-based editing, particularly with single-stranded donor oligonucleotides, added another route for targeted DNA editing [32]. Work on Cas12a-induced DNA breaks clarified why precise repair outcomes vary and how donor-based repair might be improved [97]. Later studies enabled defined substitutions, insertions, and deletions at selected loci [34]. Co-targeting supported scarless editing of genes without obvious selectable phenotypes [98], and a targeted insertional mutagenesis method combined Cas9 ribonucleoproteins with donor DNA to generate targeted insertions [99]. A recyclable marker system made repeated oligonucleotide-directed editing more practical in one genetic background [100]. Studies of epigenetic transgene silencing and CRISPR-based disruption of epigenetic regulators also identified host pathways that act on foreign DNA and improved transgene-expression stability [37,85]. These studies connect editing chemistry, repair behavior, and expression stability in one model chassis.

Editing tools in *P. tricornutum* have developed together with improvements in DNA delivery and vector maintenance. Using particle bombardment-mediated delivery, targeted genome engineering was first demonstrated with meganucleases and transcription activator-like effector nucleases for *P. tricornutum* [39]. The later development of diatom episomes delivered by bacterial conjugation enabled CRISPR/Cas9 as a more flexible editing system, allowing recovery of transgene-free edited lines and multiplexed knockouts [40,101,102]. Besides, DNA-free Cas9 ribonucleoprotein delivery enabled one-step generation of double- and triple-knockout strains [41]. Thus, the strength of *P. tricornutum* editing lies not only in nuclease activity but also in the combination of programmable nucleases with diatom-adapted delivery routes. One practical limitation is that *P. tricornutum* is diploid, so edited colonies may include monoallelic, biallelic, heterozygous, or homozygous configurations. Fully edited strains therefore often require careful genotyping, clone purification, and sometimes additional rounds of selection or subculturing.

For *Nannochloropsis* spp., CRISPR/Cas9 editing was first established in *N. oceanica* using nitrate reductase as a target, opening the way for routine targeted disruption in this oleaginous genus [51]. Subsequent work in *Nannochloropsis* spp. addressed the practical challenge of engineering multiple traits without accumulating selectable markers. Genome editing combined with marker recycling enabled markerless trait stacking in *N. gaditana*, whereas an episomal CRISPR/Cas9 system enabled marker-free gene disruption in *N. oceanica* [53,54]. A broader CRISPR/Cas12a-based toolbox later added multiplex editing, homology-directed repair, and gene interference [55]. The editing efforts have since expanded to include knock-in at a transcriptional hotspot for safe-harbor expression [103], deletion of large genome fragments [104], transcriptional activation [105], and base editing [106]. These tools make *Nannochloropsis* spp. increasingly suitable for engineering traits that require coordinated modification of multiple genes, including EPA biosynthesis, carotenoid production, and carbon partitioning.

Editing workflows can also be made conditional. One approach is to limit the duration or inheritance of active Cas nucleases. In *C. reinhardtii*, inducible Cas protein expression improves genome-editing workflows [107]. In *P. tricornutum*, conjugation-delivered episomes can introduce editing machinery and then be removed to obtain transgene-free edited lines [101]. In *Nannochloropsis* spp., ribonucleoprotein-mediated homology-directed repair and episomal CRISPR/Cas9 systems provide related routes toward cleaner and more controllable editing outcomes [53,54]. Another approach is to use catalytically inactive Cas proteins for transcriptional control rather than DNA cutting. CRISPRi and CRISPRa have been reported in *N. oceanica*, where they repress or activate target genes [55,105]. These approaches add reversible control to a field still dominated by knockout workflows.

### From genetic tools to iterative engineering workflows

The tools discussed above become most useful when they can be combined in iterative strain-engineering cycles. A DBTL workflow asks a simple practical question: Does one round of engineering generate information that makes the next round better designed? For microalgae, this is still challenging [108]. A useful cycle usually starts with a specific variable, such as a promoter series, targeting peptide, insertion site, or inducible regulatory module. Instead of selecting one successful transformant, the build step should generate a comparable set of strains. Testing then needs to measure the desired output such as growth or product content, together with expression stability, clone-to-clone variation, cultivation state, light and carbon conditions, and physiological burden. The most useful outcome is not simply a better-performing clone, but information that explains how the next construct or strain design should be improved.

Such iteration is routine in bacteria and yeast, where library construction, barcoded tracking, automated phenotyping, and computational design tools can be combined across many design cycles [109,110]. By contrast, microalgae are less standardized: Transformation throughput is relatively low, culture conditions strongly shape phenotypes, and compartmentalized metabolism makes across-study comparison challenging [108]. Recent algal work nevertheless points in the right direction. Machine-learning-informed semi-continuous cultivation has linked growth data with culture management, and reproducibility-focused work in *C. reinhardtii* has shown how temperature, pH, nutrients, culture density, and gas exchange affect experimental outcomes [111,112]. For microalgal chassis development, the immediate need is not only better strains but better experiments: Each round should report enough genetic, physiological, and cultivation information for each experiment to inform the next one. Figure 2 integrates cell access, expression design, genome editing, and phenotype evaluation into a single iterative engineering workflow, highlighting where DBTL cycles most often break in the 3 reference chassis.

**Fig. 2.**
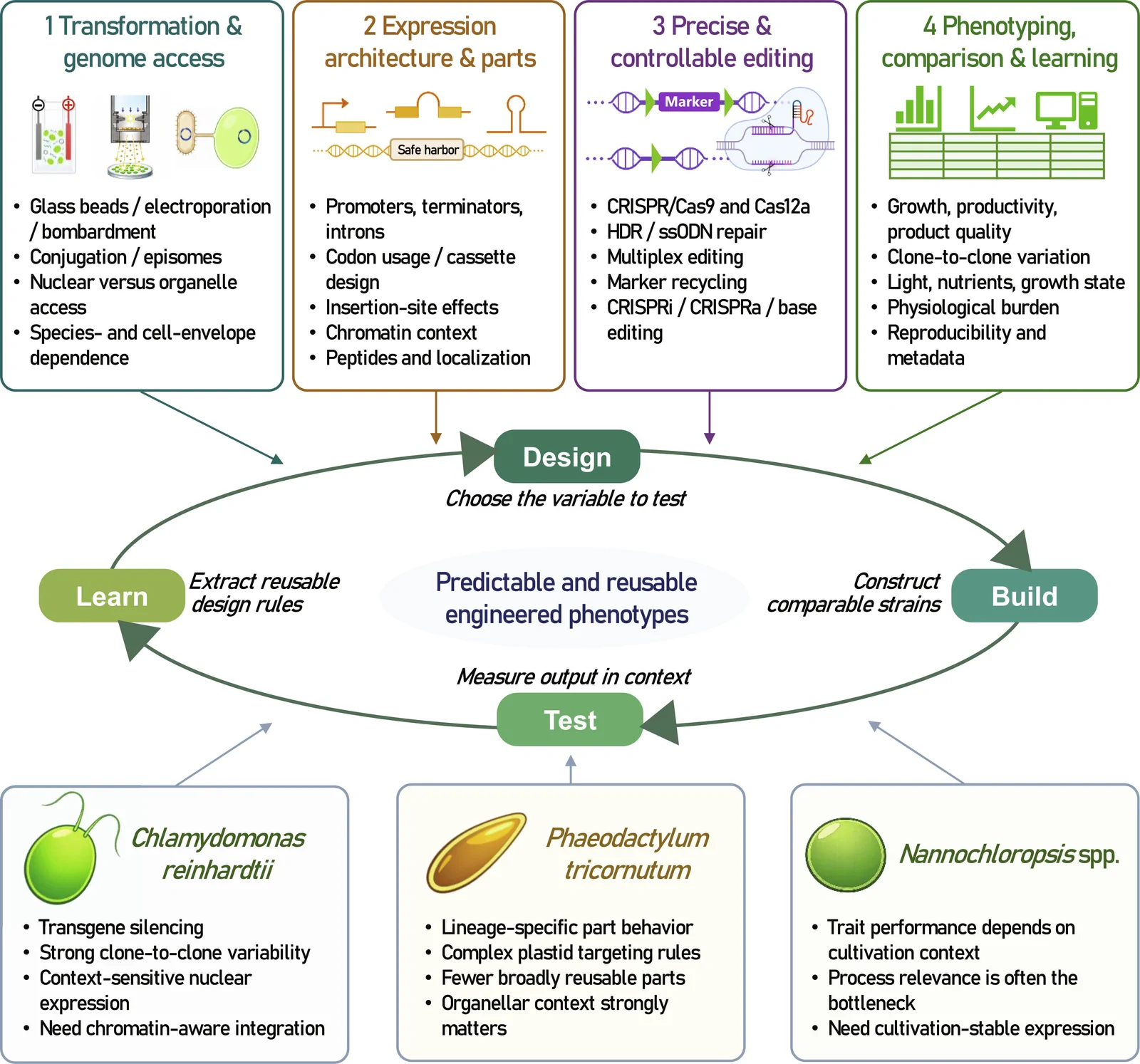
From enabling tools to an iterative DBTL workflow in microalgal synthetic biotechnology. The figure integrates the 4 enabling layers emphasized in this review: transformation and genome access, expression architecture and parts, precise and controllable editing, and phenotype evaluation within a DBTL framework. The central scheme highlights that these capabilities become useful only when they function as a connected engineering cycle rather than as isolated methods. The lower panel summarizes representative failure points that currently limit iteration in the 3 reference chassis. These are representative constraints rather than chassis-exclusive limitations.

## Trait Programs for Evaluating Chassis Performance

A synthetic biology toolbox becomes most valuable when it can improve traits repeatedly and under conditions close to real use. In microalgae, 4 trait areas now provide useful tests of chassis performance: photosynthetic productivity and stress tolerance, storage lipids and omega-3 PUFAs, terpenoids and carotenoids, and pollutant removal. These areas differ in application, but they raise a common question: Can a genetic intervention be translated into a stable physiological phenotype that remains relevant beyond a laboratory assay?

### Photosynthetic productivity and stress tolerance

In dense microalgal cultures, greater light capture does not necessarily translate into higher productivity. Cells near the light source can receive more photons than they can use, whereas cells deeper in the culture become light limited. This uneven light distribution increases self-shading and can reduce culture-level productivity [113]. Photosynthetic engineering therefore has to balance light harvesting, photoprotection, carbon-concentrating mechanisms (CCMs), redox state, and carbon sinks [4,114–116].

In *C. reinhardtii*, productivity-oriented studies are mainly related to light use and photoprotection. Reducing photosystem antenna size can improve light distribution and photosynthetic productivity in dense or high-light cultures [117]. Engineering astaxanthin accumulation can likewise reduce photoinhibition and increase biomass productivity under high light [118]. In addition, studies on CCMs and the pyrenoid of *C. reinhardtii* contribute to the understanding of eukaryotic carbon fixation [119–122]. The pyrenoid is a chloroplast structure where Rubisco is concentrated through phase-separated organization [114,123]. This mechanism has also provided insights into transferring microalgal CCM into plant chloroplasts [124]. Thus, *C. reinhardtii* is valuable both as an engineering system for testing light-use efficiency and high-light tolerance, and as a mechanistic model for understanding how eukaryotic microalgae organize CO_2_ delivery around Rubisco for bio-fixation.

In *P. tricornutum*, photosynthetic engineering has often been related to carbon metabolism and photoprotection. One example comes from the overexpression of a mitochondrial phosphoenolpyruvate carboxylase, which increased the microalgal photosynthetic productivity as well as biomass production [125]. A second example is the study of light-harvesting complex stress-related proteins (Lhcx), which could be engineered to confer photoprotection on *P. tricornutum* [126]. These examples point to 2 practical entry points for diatom photosynthetic engineering: enhancing carbon fixation and protecting against photodamage from excess excitation energy.

The *Nannochloropsis* examples are more application-facing. In *N. oceanica*, overexpression of a Rubisco activase gene was able to improve photosynthesis, growth rate, and biomass productivity under air-level CO_2_ [127]. *Nannochloropsis* CCMs involve a set of carbonic anhydrases. Knockdown of a cytosolic carbonic anhydrase promoted biomass accumulation under high CO_2_ [128]. More recently, knockout of a chloroplastic aldolase/Rubisco lysine methyltransferase gene showed that biomass accumulation could be achieved under high light, partly by alleviating photoinhibition and improving carbon fixation [129].

For all 3 chassis, photosynthetic engineering improves performance only when extra absorbed light or fixed carbon becomes a stable growth or production trait. Otherwise, the added input can disappear through heat dissipation, photodamage, or metabolic imbalance.

### Storage lipids and omega-3 PUFAs

Lipid engineering has become one of the most active areas of microalgal biotechnology. In many algae, storage-lipid accumulation is closely linked to slower cell division, nutrient stress, membrane acyl remodeling, and changes in cellular energy balance [44,130–132]. Recent studies have moved lipid engineering away from descriptive stress-induced accumulation and toward more defined steps, including acetyl-coenzyme A (CoA) supply, glycerolipid assembly, nitrogen-responsive carbon allocation, and lipid-class remodeling [15,17,133]. Within this field, *C. reinhardtii* is still especially valuable for resolving regulatory mechanisms, whereas *P. tricornutum* and *Nannochloropsis* spp. are more directly linked to lipid-product design and product-driven engineering [16,21].

TAG has received the most attention as a target of storage-lipid engineering. In microalgae, TAG is formed mainly through 2 pathways: the acyl-CoA-dependent Kennedy pathway and the acyl-CoA-independent pathway mediated by phospholipid:diacylglycerol acyltransferase (PDAT) [134]. It has been reported that manipulating key enzymes involved in these pathways, including glycerol-3-phosphate acyltransferase (GPAT), lysophosphatidic acid acyltransferase (LPAAT), diacylglycerol acyltransferase (DGAT), or PDAT, could improve TAG accumulation in several microalgae [135–142]. In many cases, the engineered TAG increases are stronger under nutrient-replete or moderately stressful conditions than under nutrient-deprived conditions [15,17]. This pattern suggests that acyltransferase engineering often changes the timing, flux, or lipid-class partitioning of TAG synthesis, but does not replace the broader stress physiology that normally drives storage-lipid accumulation.

Engineering omega-3 PUFAs raises constraints that differ from storage-lipid engineering. EPA and related long-chain PUFAs are not only bulk storage compounds. In many microalgae, they are enriched in membrane polar lipids, and their abundance can decrease when cells enter stress states that favor neutral-lipid accumulation [59]. For this reason, an EPA-rich strain cannot be evaluated only by total lipid percentage. The relevant questions are where EPA is synthesized, which lipid classes retain it, how efficiently it reaches harvestable fractions, and whether the final oil remains chemically stable [143,144]. *Nannochloropsis* spp. and *P. tricornutum* have become reference systems for PUFA engineering, because both possess native LC-PUFA metabolism, are relevant to EPA production, and have versatile engineering tools [20,21,48,145].

*Nannochloropsis* spp. represents a well-studied host to show how EPA engineering is shifting from pathway overexpression toward control of flux and lipid routing. EPA biosynthesis occurs largely in the endoplasmic reticulum and involves a set of fatty acid desaturases and elongases [49]. Poliner and colleagues [52] tried to overexpress several endogenous fatty acid desaturase genes in *N. oceanica* individually or in combination, but the EPA increase was only marginal and gene-dependent. Later work identified a Δ6 fatty acid elongase that is involved in the omega-6 route for EPA biosynthesis [146], developed a pull-and-push strategy by combining elongase and EPA-preferred DGAT manipulation to enrich EPA in TAG [59], and combined desaturase/elongase modules with storage-lipid engineering to improve both EPA accumulation and partitioning [147]. More recent studies have clarified that *N. oceanica* mainly employs the omega-6 route to synthesize EPA, and that relieving an early elongation bottleneck through NoELO2 overexpression can substantially increase EPA without compromising growth [61,62,148]. It should be noted that the synthesized EPA needs to be transferred into chloroplastic membranes [49]. Therefore, engineered EPA production is impacted not only by precursor supply, biosynthesis capacity, and routing but also by the storage destination.

The design goal of lipid engineering depends strongly on the intended lipid product. For fuels and bulk oleochemicals, lipid content, biomass productivity, harvesting cost, and process simplicity remain major considerations [133]. If PUFAs such as EPA are the target, the EPA content, lipid-class composition, oxidative quality, and regulatory traceability all matter [143]. This distinction is particularly important for *Nannochloropsis* spp., because the genus combines oleaginous growth with native EPA metabolism [21]. By contrast, *C. reinhardtii* remains more useful for mechanism-centered studies than for direct lipid production [134].

### Terpenoid and carotenoid engineering

Microalgae are promising sources of terpenoids and carotenoids, many of which have high commercial value [63,64,149–151]. Their engineering, however, may be constrained by microalgal physiology, as many carotenoids participate in light harvesting, photoprotection, membrane organization, or stress responses. Of the 3 chassis, *C. reinhardtii* has been widely used for testing heterologous terpenoid biosynthesis and ketocarotenoid engineering [118,152–158], *P. tricornutum* is especially relevant to fucoxanthin biosynthesis and engineering [64,159–161], while *Nannochloropsis* spp. has emerged for engineering ketocarotenoid biosynthesis [60,162].

In *C. reinhardtii*, the well-characterized green algal background makes pathway behavior easier to test. Early work demonstrated the feasibility of using *C. reinhardtii* for light-driven production of the sesquiterpenoid patchoulol [154]. Later studies extended production to (E)-α-bisabolene, nootkatone, sclareol, casbene, taxadiene, and manoyl oxide [155–158,163,164]. Carotenoid engineering in *C. reinhardtii* has made progress, for example, reviving or redesigning ketocarotenoid pathways to enable astaxanthin accumulation [118,152,153], redirecting carotenoid flux to enhance astaxanthin production [165], and engineering multiple pathways to enable high-yield zeaxanthin production [166]. These examples use the model chassis to examine how light, carbon supply, pathway design, and product metabolism interact.

The carotenoid profile of *Nannochloropsis* spp. differs from that of *C. reinhardtii*, with violaxanthin, vaucheriaxanthin, and β-carotene as the major components [167]. Overexpressing the endogenous lycopene β-cyclase (LCYB) gene increased β-carotene levels in *N. oceanica* without impacting algal growth [167]. Introducing heterologous β-carotene ketolase (BKT) and β-carotene hydroxylase (CHYB) enabled *N. oceanica* to accumulate ketocarotenoids such as canthaxanthin and astaxanthin [60,162]. Specifically, BKT and CHYB had to be targeted to the chloroplast to access the direct precursor β-carotene. Astaxanthin accumulation increased further when carotenoid biosynthesis was pushed by overexpressing endogenous geranylgeranyl diphosphate synthase (GGPPS), carbon flux was redirected from violaxanthin to astaxanthin by suppressing zeaxanthin epoxidase (ZEP), or astaxanthin storage was promoted by expressing a heterologous orange carotenoid binding protein [60]. Astaxanthin accumulation also allowed *N. oceanica* to grow better and produce more EPA under high-light stress, linking product formation with stress robustness in one chassis [60].

Terpenoid and carotenoid engineering shows that pathway installation is only the first step. A useful pigment phenotype also depends on precursor supply, enzyme localization, storage capacity, and the physiological role of the compound in the host cell. Notably, carotenoids are photosynthetic pigments and their engineering can alter photosynthetic physiology. The main design problem is to raise product formation while preserving photosynthetic and cultivation performance.

### Wastewater treatment and engineered pollutant removal

Wastewater applications test a different boundary of chassis engineering. Microalgae already remove nitrogen and phosphorus and contribute to the uptake or transformation of pollutants, particularly when grown together with bacteria. However, treatment performance often varies with wastewater source, season, light conditions, reactor design, and microbial community composition [168–172]. In this context, synthetic biotechnology is most valuable when it introduces a specific catalytic or sensing function, helps engineered strains remain effective under changing operating conditions, or enables process performance to be monitored more reliably.

Pollutants that are not removed efficiently by uptake alone may require an introduced catalytic activity. Cytochrome P450 provides one example and has been introduced into microalgae to remove pharmaceutical contaminants, antibiotics, and nitrogen from livestock and poultry wastewater under continuous cultivation [169,173]. These studies tested a defined catalytic function in a photosynthetic chassis under conditions closer to a treatment process. Engineered biosensors are less mature, but they may help when wastewater composition changes faster than endpoint chemical analysis can guide operation [174]. Their near-term role is likely to be process diagnosis or participation in sensing-remediation consortia.

In realistic wastewater systems, the engineered microalga is only one member of a managed community. Bacteria contribute to organic-matter degradation, nutrient cycling, and breakdown of compounds that algae cannot process efficiently [175–177]. Field-oriented remediation will therefore require compatibility among the engineered trait, algal–bacterial interactions, reactor management, biomass recovery, and containment. The 3 chassis remain valuable as reference systems for tool development and mechanistic studies, whereas practical deployment will depend on strains and consortia adapted to local wastewater conditions and regulatory requirements.

## From Engineered Phenotypes to Reusable Platforms

Microalgal synthetic biotechnology has led to many engineered phenotypes, but fewer reliable platforms. Phenotype strength often depends on strain background, insertion site, selected clone, culture condition, or sampling time [2,111,178–181]. The central challenge is therefore no longer whether a trait can be engineered, but whether it remains reproducible, physiologically stable, and relevant under conditions closer to use. As summarized in Fig. 3, the 4 trait programs discussed above encounter a recurring set of filters involving intracellular pathway organization, expression stability, process translation, and the biosafety requirements of the intended application.

**Fig. 3.**
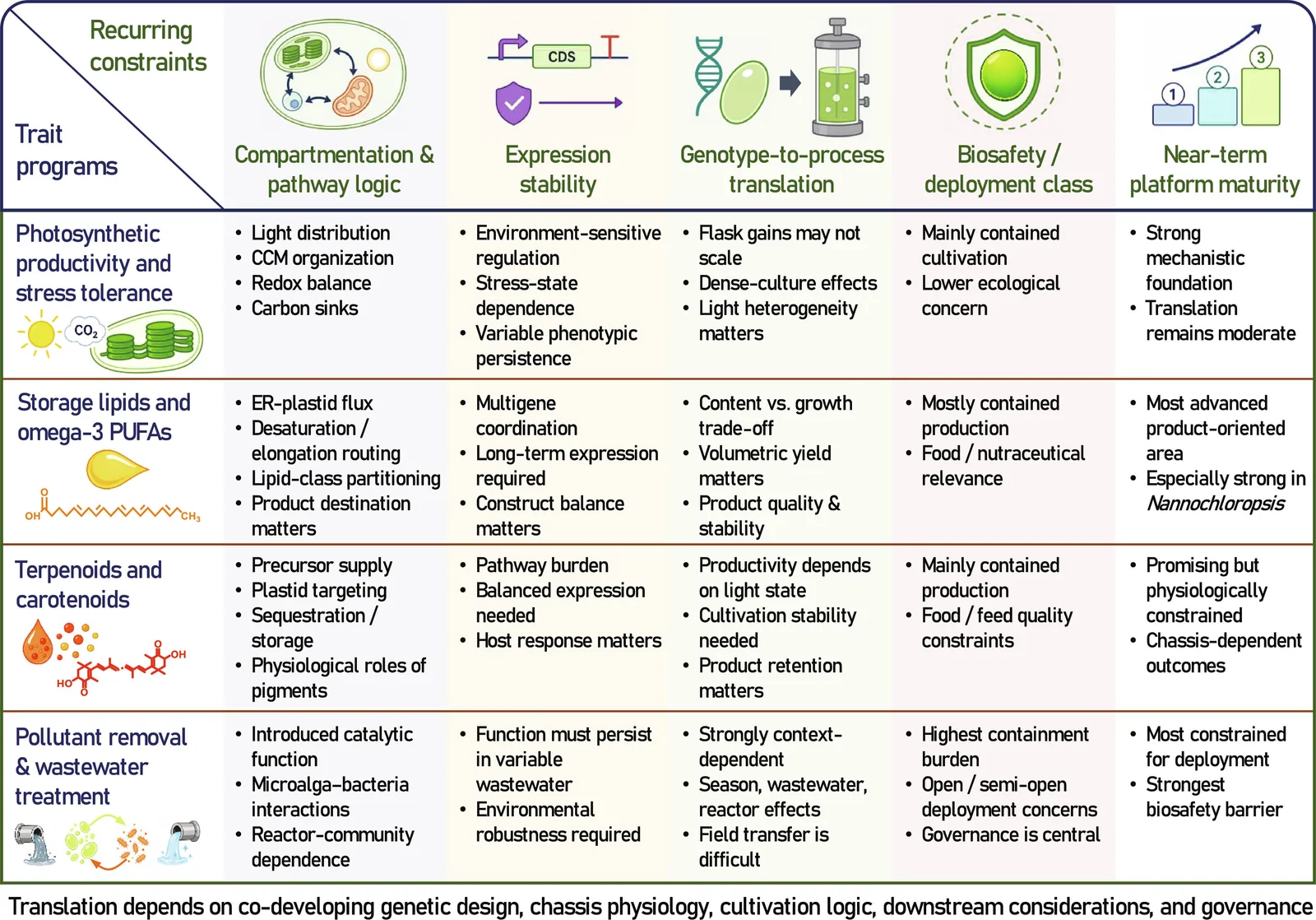
Trait programs encounter recurrent deployment filters across microalgal synthetic biotechnology. The matrix maps the 4 representative application programs discussed in the review, namely, photosynthetic productivity and stress tolerance, storage lipids and omega-3 polyunsaturated fatty acids, terpenoids and carotenoids, and pollutant removal, against 4 recurring constraints that shape translation beyond proof-of-concept: compartmentation and pathway logic, expression stability, genotype-to-process translation, and biosafety or deployment class. The rightmost column summarizes what these constraints imply for near-term platform maturity in each application area.

### Transgene silencing, expression stability, and part portability

Among the 3 chassis, *C. reinhardtii* most clearly shows that an extensive genetic toolkit does not guarantee reliable transgene expression. Nuclear transgenes are often weakly expressed, vary substantially among transformants, or become less active after prolonged cultivation [37]. One important cause is epigenetic silencing of foreign DNA. The study of mutant strains UVM4 and UVM11 has shown that loss of a sirtuin-type histone deacetylase can reduce this epigenetic repression, allowing high expression of transgenes in *C. reinhardtii* [85]. Disruption of additional epigenetic regulators can also improve transgene expression in the microalga [37]. In addition, expression can be improved through codon optimization, the inclusion of suitable endogenous introns, and the use of well-matched promoters and terminators [33,79,80]. Nevertheless, random integration still creates strong position effects because of the cassette structure, insertion site, and copy number [83]. Targeted integration into validated genomic loci or landing pads should therefore provide greater stability than screening large numbers of random transformants.

Transgene silencing has been less extensively studied in *P. tricornutum* and *Nannochloropsis* spp. [182]. Both chassis can support stable genomic expression, but substantial differences between independent transformants are still common. Such differences are generally associated with random integration, copy number, and promoter–gene combinations rather than with a well-defined silencing mechanism. This issue can be partly addressed by using episomal systems in *P. tricornutum* and targeted integration in *Nannochloropsis* spp. [42,56,183].

The limited portability of genetic parts also restricts microalgal engineering. Genetic parts are often characterized using only one reporter, strain, or growth condition, and not readily transferable between green algae, diatoms, and eustigmatophytes [33,57]. The priority is therefore not simply to expand part libraries, but to benchmark existing parts under standardized conditions. Comparing parts at defined genomic loci, across species and growth states, and over extended cultivation would help define their operating ranges and support more reliable reuse in different constructs and chassis.

### From laboratory phenotypes to process performance

Laboratory assays are essential for identifying the effects of genetic engineering, but the traits measured in small batch cultures do not always translate into useful process performance. Endpoint biomass, cellular pigment content, or lipid accumulation after short-term nutrient stress may reveal a clear biological response, yet provide little information about sustained productivity or product recovery [111,178,181,184]. This limitation is particularly relevant to lipid or carotenoid engineering. Severe nutrient limitation can markedly increase TAG or astaxanthin content while reducing cell growth [59,151], so a higher cellular content may not lead to a higher volumetric yield. Similarly, a growth advantage observed in a dilute flask culture may disappear in dense cultures or scaled-up culture systems [185].

Engineered microalgal strains should therefore be evaluated over cultivation periods and operating conditions that reflect their intended use. Harvesting, extraction, and product stability also need to be considered when they affect the practical value of the engineered trait. Standardized reporting of cultivation and downstream parameters would enable more meaningful comparisons between studies and help determine whether a laboratory phenotype can be maintained under process-relevant conditions [185].

### Biosafety and regulation beyond contained cultivation

Biosafety becomes a more immediate concern when engineered microalgae are used outside fully contained facilities. In outdoor ponds, semi-open reactors, or wastewater systems, assessment must consider whether the strain can escape, remain in the environment, exchange genetic material, or alter the surrounding community [168,186,187]. The level of concern also depends strongly on the final application of the engineered strains. Producing a purified compound in a closed facility is different from using living engineered cells in wastewater treatment or environmental remediation. The latter requires closer attention to persistence, dispersal, and ecological effects [188,189]. An effective strain may still have little practical value if it cannot be reliably contained, detected, or removed.

Public response is also likely to depend on how the engineered organism is used. A purified product made in a controlled process is unlikely to raise the same concerns as the release of viable cells into an open environment. Clear evidence of benefit, together with practical containment and monitoring measures, can make the risks easier to assess and communicate [190,191]. Biosafety and regulation should therefore be considered during strain and process development, rather than addressed only after a promising phenotype has been obtained.

### Evaluating microalgal chassis maturity

A mature chassis should support repeated strain construction without requiring extensive screening each time. Transformation and editing need to work consistently, and similar constructs should give comparable expression across independent lines and successive passages. Genetic parts should also remain functional when light, nutrients, or growth phase change. Equally important, the engineered trait must be compatible with the physiology of the host. A strain that produces more of a target compound but grows poorly or loses photosynthetic capacity may still be useful for studying mechanisms, but it is unlikely to serve as a robust production strain. For applications outside the laboratory, cultivation performance, product recovery, containment, and regulatory requirements also need to be considered [168,185,192]. The main limitations and immediate priorities of microalgal platforms are summarized in Table 2.

**Table 2.** Cross-cutting bottlenecks, minimum evidence, and near-term priorities for deployable microalgal platforms

Bottleneck	Why it matters	Minimum evidence or benchmark	Near-term priority
Expression stability and chromatin context	Weak, variable, or declining expression increases clone screening and prevents reliable reuse across engineering cycles	Multiple independent lines; expression and phenotype tracked over passages; insertion site, copy number, and cassette structure verified; performance tested in defined genomic contexts	Validate safe harbors and landing pads; develop chromatin-aware cassette designs; establish inheritance and long-term expression benchmarks
Part portability and environmental context	Promoters, introns, terminators, and targeting signals can change behavior with strain background, cassette pairing, light, nutrients, or growth state	Parts compared at defined loci with more than one construct and under multiple growth states; dynamic range, variability, and failure conditions reported	Build chassis-specific but benchmarked promoter, intron, terminator, and targeting libraries using shared reporting standards
Editing precision, repair control, and iterative engineering	Unpredictable repair outcomes and repeated marker use hinder pathway reconstruction, multiplex modification, and trait stacking	Full-locus genotyping; editing efficiencies reported across loci; structural variants and unintended integrations assessed; marker recycling or marker-free editing demonstrated	Improve HDR and repair control; standardize genotyping; expand marker-light workflows and validated safe-harbor sites
Compartment-aware pathway organization	Mislocalized enzymes, transport limitations, cofactor imbalance, and inappropriate product sinks can obscure the true pathway bottleneck	Experimental localization; organelle-resolved metabolite or flux measurements; lipid-class or product partitioning; perturbation and rescue evidence where feasible	Combine targeting validation with transport, cofactor, and sink engineering, supported by organelle-resolved pathway analysis
Genotype-to-process translation and evaluation standards	A flask or endpoint phenotype may disappear at higher density or scale, while increased cellular content may not improve volumetric productivity or recoverable yield	Time-course growth and volumetric productivity; product composition and stability; testing under relevant light, CO_2_, nutrient, density, and reactor conditions; downstream recovery considered	Develop trait-specific scorecards and standardized cultivation metadata; co-design strain engineering with cultivation and downstream processing
Biosafety, containment, and deployment boundaries	Open or semi-open use raises application-specific concerns about escape, persistence, gene transfer, ecological effects, and public acceptance	Application-specific risk assessment; containment efficacy; strain detectability and removal; environmental persistence; post-use biomass management evaluated	Incorporate biocontainment and monitoring during strain development; define clear pathways for contained versus environmental deployment

HDR, homology-directed repair

The 3 chassis reviewed here have developed along different paths. These differences do not establish a simple ranking. Instead, they determine which chassis is best suited to a particular question or application. Across all 3, however, further progress depends on improving the predictability of expression, the interpretability of engineered phenotypes, and the connection between strain design and practical performance.

## Outstanding Questions and Perspectives

Microalgal synthetic biotechnology has already delivered convincing examples in photosynthetic engineering, lipid and EPA production, carotenoid and terpenoid synthesis, and pollutant removal. The more difficult question is why many of these results are still hard to reproduce, extend, or carry into practical cultivation.

One reason is that expression remains strongly dependent on cellular and genomic context. For most chassis, we still do not know enough about which loci support stable expression, which regulatory elements remain active as growth conditions change, or which targeting sequences work reliably for a given organelle. These issues often become apparent only after a construct is moved to another clone, combined with a larger pathway, or tested outside the original culture condition. Better-defined insertion sites, expression rules, and localization signals would therefore make later engineering less dependent on repeated trial and error.

The way engineered strains are evaluated also needs to change. A high-performing clone or a strong endpoint phenotype is useful for showing that a design can work, but it says little about how reproducible or durable that design will be. More informative studies would follow the phenotype over time and examine how it affects growth, product composition, and overall productivity. This is especially important when an increase in cellular product content is accompanied by slower growth or reduced physiological stability.

The intended application should also shape the engineering strategy from the beginning. For strains used to produce purified pigments or nutritional oils in contained facilities, the main concerns are stable production, product quality, recovery, and traceability. Live engineered cells used in wastewater treatment or environmental remediation raise additional questions because their performance depends not only on the strain itself but also on reactor operation, wastewater composition, and interactions with other microorganisms. Containment and post-use biomass management then become part of the design problem rather than separate regulatory considerations.

The comparison of *C. reinhardtii*, *P. tricornutum*, and *Nannochloropsis* spp. does not point to a single chassis that can serve every purpose. Instead, it shows where more basic platform work is still needed. Promoters must be tested beyond one growth condition, candidate insertion sites need to be examined with different constructs, and targeting sequences should be validated experimentally rather than assumed to be transferable. Improvements in editing and phenotyping are equally important. Such advances may attract less attention than a new high-titer strain, but they determine whether later strains can be built more reliably and whether one study can genuinely inform the next.
